# How phloem-feeding insects face the challenge of phloem-located defenses

**DOI:** 10.3389/fpls.2013.00336

**Published:** 2013-08-29

**Authors:** Torsten Will, Alexandra C. U. Furch, Matthias R. Zimmermann

**Affiliations:** ^1^Institute of Phytopathology and Applied Zoology, Centre for BioSystems, Land Use and Nutrition, Justus-Liebig-UniversityGiessen, Germany; ^2^Institute of General Botany, Justus-Liebig-UniversityGiessen, Germany

**Keywords:** aphid-plant interactions, aphid saliva, P-proteins, plant defense, phloem, sieve element occlusion

## Abstract

Due to the high content of nutrient, sieve tubes are a primary target for pests, e.g., most phytophagous hemipteran. To protect the integrity of the sieve tubes as well as their content, plants possess diverse chemical and physical defense mechanisms. The latter mechanisms are important because they can potentially interfere with the food source accession of phloem-feeding insects. Physical defense mechanisms are based on callose as well as on proteins and often plug the sieve tube. Insects that feed from sieve tubes are potentially able to overwhelm these defense mechanisms using their saliva. Gel saliva forms a sheath in the apoplast around the stylet and is suggested to seal the stylet penetration site in the cell plasma membrane. In addition, watery saliva is secreted into penetrated cells including sieve elements; the presence of specific enzymes/effectors in this saliva is thought to interfere with plant defense responses. Here we detail several aspects of plant defense and discuss the interaction of plants and phloem-feeding insects. Recent agro-biotechnological phloem-located aphid control strategies are presented.

A key characteristic of higher-level plants (including monocots and dicots) is the existence of a vascular network that is composed of the phloem and xylem. The vascular system pervades the whole organism from root to shoot and distributes nutrients and water. The fact that sugars, amino acids, and other organic metabolites are available via phloem and xylem in significant amounts makes the vascular system a target for insect pests (Brodbeck et al., 1993; Gündüz and Douglas, 2009). Endogenous interference in the functioning of the vascular system may have disastrous consequences for a plant’s development. Using the interaction between plants and phloem-feeding insects as an example, offensive and defense strategies during the struggle for the valuable phloem content are explored.

## THE PHLOEM: A HIGH-DENSITY ENERGY PATHWAY

In higher-level plants, a long-distance transport system has evolved to translocate photoassimilates from a source (e.g., mature leaves) to a sink (e.g., roots) – the phloem (Schulz, 1998). The angiosperm phloem is composed of sieve elements (SEs), companion cells (CCs), and phloem parenchyma cells (PPCs; van Bel, 1996; Hafke et al., 2005). Mature SEs are elongate cells lacking certain cellular components (nucleus, vacuoles, ribosomes, and dictyosomes) but lined parietally with a thin mictoplasmic layer consisting of an endoplasmic reticulum (ER), plastids, a few inactive mitochondria and phloem-specific proteins (P-proteins; van Bel, 2003). The CCs maintain SEs viability (van Bel, 2003). A high density of pore-plasmodesma units (PPUs) and tight ER coupling between SE and CC underline an intimate symplastic connection across this boundary; the entire connection constitutes the SE-CC complex (Kempers et al., 1998; Martens et al., 2006). The walls between the single SE-modules are transformed into sieve plates, perforated by plasmodesmata (PD) modified into sieve pores with a diameter up to 2.5 μm (Behnke and Sjolund, 1990; Schulz, 1998; van Bel, 2003; Mullendore et al., 2010). These adaptations provide the basis of formation of long sieve tubes based upon single SE-modules, forming a tube-like symplastic continuum that serves translocation. The complex process of this translocation is regulated via highly active CCs (van Bel and Knoblauch, 2000).

In general, the vascular system is a pressure system made up of two components (phloem and xylem) that effects the long-distance translocation of very heterogeneous constituents within higher-level plants. Xylem and phloem are parallel orientated vascular tissues in which pressure and tension gradients are built up in SEs and xylem vessels, respectively. In intact plants, the negative hydrostatic potential in xylem vessels is in balance with that inside SEs (e.g., Zimmermann et al., 2013). The driving forces for translocation are, on the one hand, a longitudinal (axial) pressure gradient within phloem and xylem, and on the other, a lateral (radial) pressure gradient between the phloem and xylem (Will and van Bel, 2006). The balanced interaction between phloem and xylem is a basic requirement for long-distance transport (van Bel, 2003; Dinant and Lemoine, 2010). The longitudinal pressure gradient within the xylem is the result of water uptake in the root/rhizosphere and loss of water by transpiration. According to the classic Münch concept (Münch, 1930) for phloem translocation, photoassimilates are amassed in the sieve tubes of source areas and escape from the sieve tubes in sink areas. The resulting turgor difference between source and sink drives the mass flow. Hence, in contrast to the xylem, the phloem exhibits a bidirectional translocation, as sink regions are found in the root and in the apex of the shoot (van Bel, 2003; Dinant and Lemoine, 2010).

**FIGURE 1:**
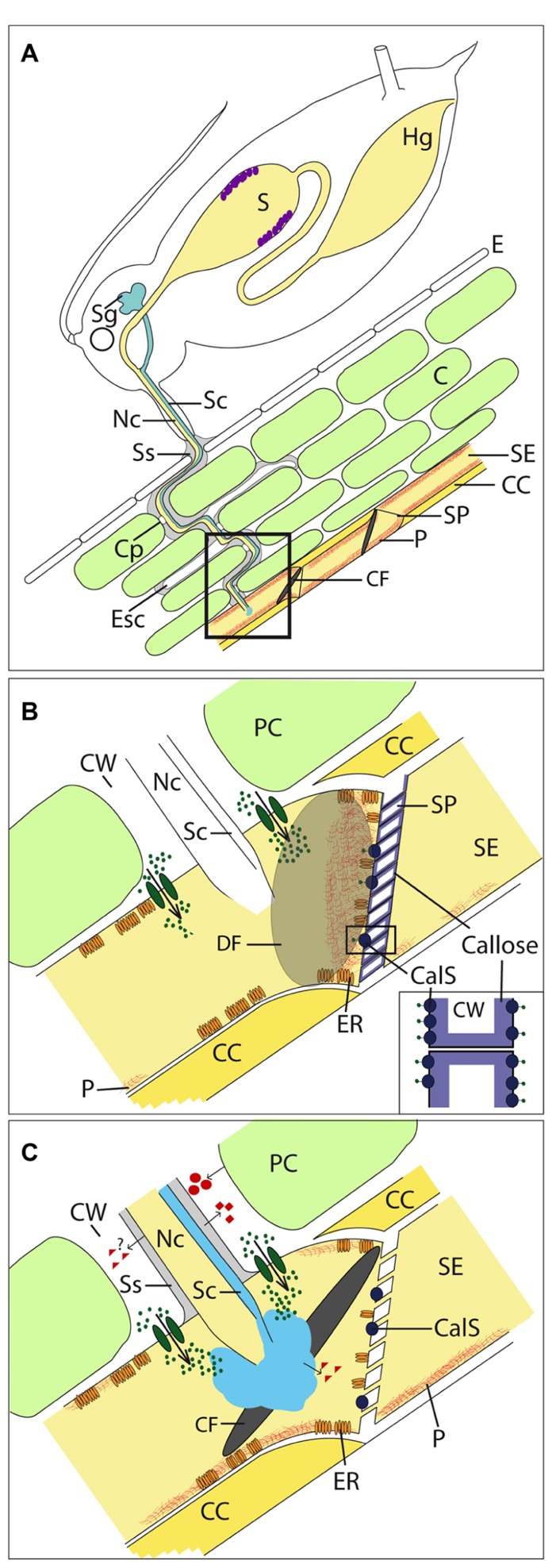
**Interaction of plants and aphids.** **(A)** Aphids penetrate the plant with their stylet and move it through the apoplast toward the sieve tubes. The stylet contains a salivary (Sc) and nutrition channel (Nc). Before plant penetration and during stylet movement, aphids secrete gel saliva (gray), which forms a salivary sheath (Ss) around the stylet. The Ss remains in the plant’s apoplast after stylet retraction and show empty stylet canals (Esc). After penetrating a sieve tube, aphids secrete watery saliva (light blue) prior to ingestion. Both saliva types are produced in the salivary glands (Sg). Proteases (violet dots) located in the stomach (S) are assumed to digest phloem-sap proteins. **(B)** The sieve tube penetration of the stylet without secretion of any saliva type would activate mechano-sensitive Ca^2+^ channels (dark green ovals) in the plasma membrane of sieve elements (SE). The consequence is a Ca^2+^ influx (dark green dots) from the apoplast and potentially from the endoplasmaic reticulum (ER) into the SE lumen. P-proteins (P, red) including the forisome and callose produced from the callose synthase (CalS; inset shows a higher magnification) lead to Ca^2+^ dependent sieve-element occlusion. **(C)** The secretion of gel and watery saliva most likely leads to an absence of wound-induced reaction of SEOs by Ca^2+^-binding. Beside Ca^2+^-binding, aphids are able to suppress further plant defense responses due to salivary effectors (red triangles). Furthermore, plant defense responses are induced in parenchyma cells (PCs), among others, by producing cell wall degradation products that act as pathogen-induced molecular patterns (red circles). Saliva proteins may act as pathogen-associated molecular patterns (red squares). E, epidermis; C, cortex; CC, companion cell; CF, condensed forisome; Cp, cell penetration; CW, cell wall; DF, dispersed forisome; Hg, hint gut; SP, sieve plate.

Phloem sap contains carbohydrates, proteins, and amino acids (**Table 1**) and makes SEs a favorite target for pathogens and pests (**Figure 1A**). An accession of SEs by pathogens/pests leads to varies impairments: (a) loss of nutrients, (b) disturbance of the translocation process, and (c) the infection by microbial pathogens (e.g., viruses, phytoplasmas, viroids; Dinant et al., 2010; Giordanengo et al., 2010). Therefore, plants have evolved a range of defense mechanisms against pathogens and pests, which in turn possess mechanisms with which to counteract these defenses.

**Table 1:** Aphid nutrition-related compounds and defense relevant factors of phloem sap.

Aphid nutrition-related compounds			
Constituent	Concentration	Species	Reference
**C – source**
Sucrose	100–1800 mM (ø300–700 mM)	*Triticum aestivum*; *Orzya sativa*; *Opuntia ficus-indica*; *Sonchus oleraceus*; *Medicago sativa*; *Salix viminalis*; *Solanum tuberosum*; *Brassica *sp.; *Plantago major*; *Plantago maritime*; *Prunus persica*; *Apium graveolens*	Rogers and Peel (1975); Hayashi and Chino (1986) Hayashi and Chino (1990); Girousse et al. (1991), Wang and Nobel (1995); Lohaus and Moellers (2000), Gould et al. (2004); Shimada et al. (2004), Pescod et al. (2007), Nadwodnik and Lohaus (2008)
Glucose	400 mM	*Opuntia ficus-indica*	Wang and Nobel (1995)
Fructose	400 mM	*Opuntia ficus-indica*	Wang and Nobel (1995)
Raffinose	70 mM	*Cucurbita maxima*	Haritatos et al. (1996)
Stachyose	330 mM	*Cucurbita maxima*	Haritatos et al. (1996)
Carbohydrates (total)	534–1800 mM (ø500–800 mM)	*Lycopersicon esculentum*; *Alonsoa meridionalis*; *Cucurbita maxima*; *Cucumis melo*	Haritatos et al. (1996); Voitsekhovskaja et al. (2006), Pescod et al. (2007); Turgeon and Wolf (2009), Zhang et al. (2010)
**N – source**
Amino acids	41–1230 mM (ø200–500 mM)	*Zea mays*; *Triticum aestivum*; *Orzya sativa*; *Opuntia ficus-indica*; *Pisum sativum*; *Medicago sativa*; *Brassica napus*; *Brassica carinata*	Fukumorita and Chino (1982); Hayashi and Chino (1986) Hayashi and Chino (1990); Ohshima et al. (1990), Girousse et al. (1991, 1996), Wang and Nobel (1995); Lohaus et al. (1998), Lohaus and Moellers (2000); Faria et al. (2007), Gattolin et al. (2008)
Proteins	76–77 μg/ml; 0.1–0.2 μg/μl	*Orzya sativa*; *Triticum aestivum*; *Opuntia ficus-indica*; *Lupinus albus*	Wang and Nobel (1995), Schobert et al. (1998), Rodriguez-Medina (2009)
		**Defense relevant factors**
Ca^2+^	35 nM to 2.5 mM	*Zea mays*; *Vicia faba*	Fromm and Bauer (1994); Furch et al. (2009)
Turgor pressure	5–20 bar	*Quercus rubrum*; *Sonchus oleraceus*; *Salix babylonica*	Hammel (1968); Wright and Fisher (1980), Gould et al. (2004, 2005)

*Only studies where phloem sap was collected via stylectomy (sieve-tube sap obtained by severing stylets of ingesting aphids) and microdissection (dissection of selected cells and subsequent analysis of cell content) were considered.*

## “PLANTS IN ACTION”: THE OCCLUSION OF SIEVE TUBES IS PROTECTIVE

Damage of sieve tubes disturbs the existing pressure conditions resulting in a turgor shift (Gould et al., 2004), which impact intracellular calcium levels and the redox state. Long-distance signaling by electropotential waves (EPWs) communicates local wounding to distant plant parts (van Bel and Gaupels, 2004; Furch et al., 2007, 2009, 2010; Zimmermann and Mithöfer, 2013). This induces the occlusion of sieve tubes via the plugging of sieve pores and can be seen as primary defense response (Knoblauch and van Bel, 1998). Sieve-tube occlusion is assumed to prevent the loss of sieve-tube sap (Evert, 1982; Schulz, 1998) and to keep phytopathogens from invading through the injured site (van Bel, 2003). Two groups of sieve-tube occlusion mechanisms can be found in plants: callose deposition and protein plugging (e.g., Will and van Bel, 2006; Furch et al., 2007).

### SIEVE-PORE CONSTRICTION BY CALLOSE: LONG-TERM MECHANISM

Callose is a linear β-1,3-glucan polymer with some 1,6 branches. β-1,3-glucans are produced as helical chains which, upon heating, form a gel. It is produced enzymatically in the presence of Ca^2^^+^ by callose synthases, located in the plasma membrane, and deposited extracellularly around sieve pores (**Figure 1B**) and PD in the form of collars (Blackman and Overall, 1998; Zabotin et al., 2002) as a reaction to chemical or mechanical stress (Kudlicka and Brown, 1997; Nakashima et al., 2003; Levy et al., 2007). The pattern and timing of callose distribution and its physiological involvement suggest that callose plays an important part in plant cell growth and differentiation. In *Arabidopsis*, 12 callose synthase genes (*CalS1–12*) have been detected. *CalS1* and *CalS10* have been shown to be responsible for cell plate formation during cell division (Hong et al., 2001; Thiele et al., 2008; Guseman et al., 2010). *CalS5*, *CalS9, CalS10, CalS11*, and *CalS12* play unique roles during microsporogenesis and pollination (Dong et al., 2005; Enns et al., 2005; Nishikawa et al., 2005; Töller et al., 2008; Huang et al., 2009; Xie et al., 2010). *CalS12* can be induced by pathogen infection and wounding (Jacobs et al., 2003; Nishimura et al., 2003; Dong et al., 2008), and CalS7 deposits callose in the phloem (**Figure 1B**; Xie et al., 2011). Xie et al. (2011) showed that callose deposition in the phloem, especially in the SEs, was greatly reduced in cs7 mutants. Callose accumulation was eliminated in the PDs of incipient sieve plates resulting in fewer sieve pores of cs7 mutants. They conclude that *CalS7* is a phloem-specific callose synthase gene, and is responsible for callose deposition in developing SEs during phloem formation and in mature phloem induced by wounding. The physiological roles of the remaining *CalS *genes in *Arabidopsis* are still unknown.

Callose deposition is a universally observed mode of sieve-plate occlusion (Kudlicka and Brown, 1997; Nakashima et al., 2003). Reversible callose accumulation apparently plays a role in regulating cell-to-cell transport through sieve pores and PPUs (Furch et al., 2007). After a heat stimulus is applied to the leaf tip of *Vicia faba* plants, callose gradually builds up at sieve plates and PD. After reaching a maximum, callose is degraded at a lower rate than production. Callose appears to degrade more rapidly at PD (30–40 min) than at the sieve pores, where the level of callose deposition reaches its original state after 1–2 h (Furch et al., 2007). β-1,3-glucan endo-hydrolases is the enzyme that catalyses callose degradation (reviewed in Leubner-Metzger, 2003). It is present in large gene families in plants (e.g., 50 genes in *Arabidopsis*) and is located in the cell membrane and highly enriched at PD sites (Levy et al., 2007). The course of callose production/degradation is qualitatively similar between different plant species, but there are variations in the time scale (Furch et al., 2007, 2008; Mullendore et al., 2010).

### SIEVE-TUBE OCCLUSION BY PROTEINS: FAST AND VERSATILE

In addition to callose, sieve pores can be blocked rapidly by proteins. In electron microscopic images, SEs show protein networks that span the SE lumen and are attached to the cell periphery (Sjölund, 1997). Sieve tubes of grasses appear virtually empty but may have an occlusion mechanism based on precipitation of soluble proteins (Will and van Bel, 2006). A specific group of phloem proteins (P-proteins) enables rapid occlusion (within some seconds) to occur in sieve tubes of higher-level plants. Numerous aggregation forms (amorphous, granular, fibrillar, filamentous, tubular, or crystalline) of P-proteins that are thought to represent stages of P-protein differentiation (Cronshaw, 1981) denote an immense variation between plant species (Cronshaw and Sabnis, 1990). The synthesis of P-proteins begins in immature, nucleate SEs, resulting in electron-dense proteinaceous structures (Ernst et al., 2012). In young SEs, subunits accumulate within the cytoplasm, forming large P-protein bodies (Steer and Newcomb, 1969). As SEs mature the P-protein bodies disperse into smaller aggregates that move to the periphery of the cell (Knoblauch and van Bel, 1998).

In cucurbits, phloem protein 1 (PP1) and phloem protein 2 (PP2) produce insoluble aggregates in response to oxidation (Kleinig, 1975; Alosi et al., 1988) by cross-linking, forming high-molecular-weight polymers that plug the sieve pores of injured sieve tubes (Read and Northcote, 1983). PP1 monomeric subunits have a predicted molecular mass of 95.4 kDa, but the apparent molecular size is dependent on the pH and oxidation state, as conformational isoforms exist that appear to be related to either the polymerized or unpolymerized, translocated forms of the protein (Clark et al., 1997; Leineweber et al., 2000). PP1 was immunolocalized in SE slime plugs and P-protein bodies, whereas the corresponding mRNA was shown to accumulate in CCs (Clark et al., 1997). Due to an interaction of PP1 and PP2 in presence of calcium and oxygen, sieve tubes and cut surfaces are rapidly occluded by gelling of the exudate (Kleinig, 1975; Clark et al., 1997; Furch et al., 2010). PP2-like proteins are lectins, sugar binding proteins, which have been identified in many angiosperms and are specifically expressed in SE/CC complexes, suggesting that PP2 may be a common component of P-proteins (Dinant et al., 2003). It has been shown to interact with phloem sap proteins, potentially playing a role in the shuttling of glycoproteins between CC and SE (Beneteau et al., 2010). The phloem-specific PP2 homolog from *Arabidopsis* was shown to be anchored to P-proteins and other phloem organelles rather than being a structural component of P-proteins (Batailler et al., 2012). These findings indicate that PP2 does not represent an essential part of the occlusion machinery. An insecticidal function for PP2 is described *in vitro* (Beneteau et al., 2010) as well as *in vivo* (Zhang et al., 2011). Hilder et al. (1995) observed that snowdrop lectin from *Galanthus nivalis*, artificially applied or expressed in *Nicotiana tabacum* plants, reduced growth, decreased survival, and lowered reproduction in the aphid species* Myzus persicae*. Other lectins that showed insecticidal effects against aphids are a mannose-binding lectin (Sauvion et al., 1996), a garlic lectin (Fitches et al., 2008), and concanavalin A (Sauvion et al., 2004). Similar effects were observed for protease inhibitors (PIs) applied to aphids via transgenic plants (Rahbé et al., 2003; Ribeiro et al., 2006; Carrillo et al., 2011). Erickson et al. (1985) observed that lectins negatively affect the activity of an aminopeptidase in rats. The identified aminopeptidase inside the aphid gut, which represents 15.6% of total gut proteins, is suggested to be a potential binding site for lectins (Cristofoletti et al., 2006).

Sieve elements of Fabaceae contain elongate protein bodies called forisomes (Knoblauch et al., 2003). Forisomes consist of fibrils (Tuteja et al., 2010) and were previously classified as “non-dispersive P-protein bodies” (Behnke, 1991). They were suspected to undergo structural transformations, from a crystalloid state with co-aligned fibrils to a “slime-body” with dispersed fibrils (Palevitz and Newcomb, 1971). The transition is a rapid and reversible conformational change in which forisomes shorten longitudinally while expanding radially with a several-fold volume increase (Knoblauch et al., 2001; Peters et al., 2007, 2008). Forisomes disperse upon wounding and occlude sieve tubes (Knoblauch and van Bel, 1998), leading to a stop of mass flow observed in artificial sieve tubes (Knoblauch et al., 2012). Furthermore, Thorpe et al. (2010) showed a cooling rate dependent transport interruption and parallel forisome dispersion in intact *V. faba* plants. Dispersion is triggered by an increase of free calcium (Ca^2^^+^) in sieve tubes (Knoblauch et al., 2001), although as of yet, no Ca^2^^+^-binding sites have been detected in forisomes. As observed *in vitro *(Knoblauch et al., 2005), high Ca^2^^+^ concentration (>50 μM) is also needed to disperse forisome *in vivo *(Furch et al., 2009). After burning the tip of a *V. faba* leaf, the elevation of Ca^2^^+^ concentration in most regions of sieve tubes inside the respective leaf was demonstrated to be below the threshold that is necessary for forisome dispersion (Furch et al., 2009). Only in the close vicinity of the Ca^2^^+^-channel pore Ca^2^^+^-level goes beyond the threshold and increases up to 100 μM (**Figure 1B**; Trewavas, 1999). Therefore, it was concluded that forisomes are directly associated with Ca^2^^+^ release sites (Furch et al., 2009). An association between forisomes and the ER (where the highest frequencies of Ca^2^^+^ channels were observed) was found. It was observed that the more intimately forisomes were associated with the ER or the plasma membrane of SEs, the greater was the probability of dispersion (Furch et al., 2009).

Scanning electron microscopic images show that forisomes are composed of largely identical subunits named forisomettes (Tuteja et al., 2010). Indicated by transmission electron microscopy studies (Ehlers et al., 2000), forisomettes consist of strictly ordered arrays of a number of forisome proteins. At least three proteins are involved in formation of forisomettes, called sieve element occlusion 1 (SEO1), SEO2, and SEO3, and were identified in *Medicago truncatula *(Noll, 2005; Noll et al., 2007). SEOs are also present in plant families that do not possess forisomes, e.g., Rosaceae, Solanaceae, and Brassicaceae (Rüping et al., 2010). Two *Arabidopsis thaliana *genes (*At3g01670 *and *At3g01680*) encode SEO proteins assigned *At*SEOR1 and *At*SEOR2 (Pélissier et al., 2008; Rüping et al., 2010; Froelich et al., 2011). Both phloem filament proteins are required for formation of filaments that are arranged as complex network inside SEs (Anstead et al., 2012). Whether the formation of dense SEO filament deposits at sieve plates stops phloem mass flow (**Figure 1B**; Ernst et al., 2012) or mass flow remains intact (Froelich et al., 2011) is a matter of debate. Aphids of the species *Myzus persicae* that feed on *At*SEOR1 and *At*SEOR2 mutants without SEO filament formation show no benefit from the absence of filaments. Thus, Anstead et al. (2012) conclude that SEOs are not involved in plant defense against phloem-feeding insects. In fact, aphids perform worse when compared to aphids on control plants, indicated by reduced reproduction and shortened reproduction period. The authors suggest that reduced fitness could be associated with lower nitrogen supply due to reduced protein content, but no data about amino acid concentration or protein content in the phloem sap of these plants are available. A further explanation could be that the absence of SEO filaments influences parameters in sieve tubes relevant for aphid feeding, e.g., turgor pressure (Miles, 1999), reducing nutrition supply and leading to the observed reduction of aphid reproduction. Although, Anstead et al. (2012) describe that the phenotype SEO mutants does not differ to the wildtype this allows no conclusion about the state of sieve tubes.

Callose deposition and protein plugging operate in parallel. A burning stimulus elicits distant occlusion in *V. faba* with rapid forisome dispersion and a slower subsequent callose deposition (Furch et al., 2007, 2009). While forisomes reconstitute into the condensed state, constriction of sieve pores by callose deposition reaches its maximum level (Furch et al., 2007). It is suggested, therefore, that plants possess a universal safety design for sieve-tube occlusion, one that proceeds rapidly and involves P-protein and a slower and more long-lasting one based on callose (Furch et al., 2007).

The distant-induced occlusion was associated with the passage of a damage-induced EPW. EPWs communicate sudden and profound physiological changes over long-distances (Furch et al., 2007; Hafke et al., 2009). EPWs trigger a release of Ca^2^^+^ that results in callose deposition and protein plugging (Kauss, 1987; Colombani et al., 2004). Ca^2^^+^ originates from the apoplast via opened plasma membrane channels or from the ER acting as an intracellular Ca^2^^+^ storage (Furch et al., 2009; Hafke et al., 2009; Zimmermann and Mithöfer, 2013).

## “INSECTS IN ACTION”: HOW PHLOEM-FEEDING INSECTS OVERWHELM PLANT DEFENSES

Phloem-feeding insects belong to the order Hemiptera. Of these, important pests are planthoppers (suborder Auchenorrhyncha) and leafhoppers (suborder Clypeorrhyncha) as well as aphids and whiteflies (suborder Sternorrhyncha). The most currently available information about interaction with plants is on aphids, which make them a model organism for phloem feeders. Phloem-feeding insects possess specialized mouthparts, so-called stylets, with which they are able to obtain nutrition from plant tissues that are located deep inside the plant (**Figure 1A**). To access their food source, phloem-feeding insects secrete saliva that potentially interacts with defense mechanisms located in the sieve tube (Tjallingii, 2006; Will and van Bel, 2006).

### THE STYLET AND ITS PATHWAY

The thin stylets of phloem-feeding insects are formed of four subunits, and their diameter and length are species dependent. The two outer mandibular parts contain nerve canals; the inner maxillary parts form the nutrition channel and a saliva channel that merge to a common duct at the tip of the stylet (Uzest et al., 2010). An insect penetrates the plant with its stylet and moves the stylet toward the sieve tubes (**Figure 1A**). The stylet moves through the apoplast without causing significant damage to plant cells (Tjallingii and Esch, 1993; Hewer et al., 2011). Plant cells of different cell types are regularly penetrated along the stylet track. Aphids take up small cell sap samples when penetrating; this sample is most likely analyzed by chemosensilla in the precibarium (Wensler and Filshie, 1969) as observed for leafhoppers (Backus and McLean, 1985). Beside acceptance of host plants (Backus and McLean, 1985) this behavior could allow aphids to orient themselves inside the plant and helps them to detect sieve tubes, whereat parameters like sucrose and pH (**Table 1**) are suggested to be indicators for sieve tube penetration (Hewer et al., 2010, 2011). After a sieve tube is penetrated, ingestion starts.

### SALIVA AND ITS RELEVANCE FOR APHID-PLANT INTERACTIONS

Before plant penetration with their stylets, during stylet movement through the apoplast, penetration of cells, and ingestion, saliva is secreted by planthoppers (Wang et al., 2008), leafhoppers (Günthardt and Wanner, 1981; Harris et al., 1981; DeLay et al., 2012), aphids (Prado and Tjallingii, 1994; Tjallingii, 2006) as well as whiteflies (Morgan et al., 2013). Saliva has been suggested to play a key role in the interaction of insect pests and their respective host plants (reviewed in Walling, 2008). Aphids in particular function as model organisms for studying both phloem-feeding insects and the role and functions of saliva inside the plant. Like other hemiptera, aphids possess two types of saliva, one gel-like and one watery (Miles, 1999), although recently the protein composition of both types was shown to overlap partly (Will et al., 2012a).

Gel saliva forms a salivary flange on the plant surface prior to plant penetration (**Figure 1A**; Will et al., 2012b), which is suggested to stabilize the stylet before initiating stylet penetration of the plant (Pollard, 1973; Tjallingii, 2006). When the stylet moves, small amounts of gel saliva are secreted; these harden and are then penetrated by the stylet (McLean and Kinsey, 1965). This continuous secretion of gel saliva leads to the formation of a solid salivary sheath that envelops the stylet and is left *in situ* after it is withdrawn from plant tissues (Will et al., 2012b). Sheath formation was assumed to be associated with the oxidation of protein sulphydryl groups, e.g., present in the amino acid cysteine (Miles, 1965; Tjallingii, 2006). Will et al. (2012a) observed in this context that salivary sheath formation is disturbed under anoxic conditions. A protein that possesses a high content of cysteine was identified in saliva of the pea aphid *Acyrthosiphon pisum* by Carolan et al. (2009) and termed “sheath protein” (SHP). It is assumed that formation of disulfide bonds leads to SHP aggregation and formation of the solid sheath. The fact that most phytophagous hemiptera were observed to form a salivary sheath during the feeding process (Morgan et al., 2013), implies biological relevance but specific functions are unknown.

It is suggested that gel saliva functions as a lubricant to facilitate stylet movement and that the sheath protects the stylet against mechanical forces and chemicals (Miles, 1999). Furthermore, Will and van Bel (2006) postulated that the salivary sheath prevents the induction of defense responses in these conduits. In contrast, some enzyme components of the gel saliva are assumed to trigger plant defense responses by forming so-called pathogen-induced molecular patterns (PIMPs; **Figure 1C**). Potential candidates for PIMP production are cell-wall-degrading enzymes, such as cellulase and pectinase, which were detected in aphid saliva (Ma et al., 1990; Cherqui and Tjallingii, 2000). Whether the protein or peptide components of gel saliva act in a similar manner to, e.g., flg22 from bacteria (Zipfel, 2008) as pathogen-associated molecular patterns (PAMPs; **Figure 1C**) that trigger plant defense responses in a gene-to-gene interaction can be speculated (Will et al., 2012b). It may be that aphid gel saliva on one hand induces plant defense in cortex cells along the stylet track and on the other hand suppresses defense inside penetrated sieve tubes (**Figure 1C**; Will and van Bel, 2008; Louis et al., 2012).

Aphid watery saliva is secreted intracellularly, either when the stylets briefly puncture cells during probing (Martin et al., 1997) or immediately before and during sap ingestion (**Figure 1C**; Prado and Tjallingii, 1994), and recent studies on *Aphis gossypii* indicate that watery saliva is secreted into the apoplast as well (Moreno et al., 2011). Gel saliva and watery saliva contain many different proteins of a broad molecular weight range (e.g., Madhusudhan and Miles, 1998; Will et al., 2007, 2009; Harmel et al., 2008; Carolan et al., 2009; Cooper et al., 2011; Nicholson et al., 2012). The main classes of proteins that were identified with a proteomic approach in the species *Acyrthosiphon pisum* are proteases, detoxifying enzymes and proteins that potentially interact with plant signaling cascades, so-called effectors (Carolan et al., 2011). Salivary proteins appear to move from the SE where they were secreted into adjacent SEs (Madhusudhan and Miles, 1998), which suggests that saliva functions are not restricted to an aphid-penetrated SE. Thus, the activity of aphids in a population puncturing SEs downstream from an SE already punctured by another aphid may be facilitated by suppressed defense responses. The aphids may therefore benefit from the saliva secretions of other individuals. The observation that feeding is locally stimulated on potato for the aphid species *Myzus persicae* and *Macrosiphon euphorbiae*, respectively, 96 h after first infestation (Dugravot et al., 2007) supports this hypothesis.

### INTERACTION WITH OCCLUSION AND SIGNALING

Sieve tubes lack most organelles and gene expression machinery but possess a variety of defense components, both physical and chemical mechanisms. Ca^2+^ represents a core of both groups (**Figure 1B**). The high concentration gradient of Ca^2+^ between apoplast and SE lumen leads to an influx of Ca^2+^ into the SE lumen during penetration of the SE membrane by a thin glass capillary, which induces occlusion (**Figure 1B**; Knoblauch and van Bel, 1998). During SE penetration by an aphid stylet this transient event is assumed to be suppressed initially by gel saliva that is secreted prior to penetration and seals the penetration site (**Figure 1C**; Will and van Bel, 2006). Walker and Medina-Ortega (2012) did not observe forisome dispersion in penetrated SEs prior to secretion of watery saliva and concluded that SE occlusion does not represent a defense mechanism against aphids, which is also suggested by Anstead et al. (2012). Nevertheless, findings of Walker and Medina-Ortega (2012) support the hypothesis that sealing of the stylet penetration site by gel saliva already mediates suppression of occlusion mechanisms (**Figure 1C**). The risk of triggering SE occlusion also occurs when aphids start to remove solute from the SE lumen, as this too potentially decreases turgor in SEs (**Table 1**) and thus activates potential mechano-sensitive Ca^2+^-channels that results, e.g., in forisome dispersion (Knoblauch et al., 2001; Furch et al., 2009). For this reason, in a second step prior to ingestion, aphids secrete watery saliva (**Figure 1C**) that contains proteins that bind Ca^2+^; these proteins were detected by functional analysis for the aphid species *Megoura viciae* and were shown to counteract SE occlusion (Will et al., 2007). Because aphids of different species change to watery saliva secretion if an occlusion event is induced during ingestion, the phenomenon of counteracting SE occlusion by secreting watery saliva is likely widespread (Will et al., 2009). *In vitro* experiments have demonstrated that this change of behavior is triggered by a decrease of turgor pressure inside the sieve tubes (Will et al., 2008) that is the consequence of SE occlusion (Gould et al., 2004).

In the saliva of *Acyrthosiphon pisum*, a Ca^2^^+^-binding protein was detected by mass spectrometry and was identified as regucalcin (Carolan et al., 2009). The molecular mass of this protein, 43 kDa, is comparable to that of a previously detected Ca^2^^+^-binding protein (Will et al., 2007). Regucalcin is a member of the senescence marker protein-30 (SMP-30) family that helps sequester signaling molecules such as Ca^2^^+^ (Fujita et al., 1992; Shimokawa and Yamaguchi, 1993). In addition, regucalcin maintains intracellular Ca^2+^ homeostasis by activating Ca^2+^ pumps in the plasma membrane, ER, and mitochondria of many animal cell types (Yamaguchi, 2000). Moreover, in animals regucalcin has an inhibitory effect on the activation of Ca^2+^/calmodulin-dependent enzymes and protein kinase C (Yamaguchi, 2005). Thus, an inhibition of signaling cascades due to Ca^2+^-binding by saliva proteins appears likely (Will and van Bel, 2006, 2008), although little information about the molecular level of defense signaling inside sieve tubes is available. A recent study by Rao et al. (2013) did not detect the presence of regucalcin in the saliva of cereal aphids and the authors suggest that different protein compositions of watery saliva of various aphid species may illustrate the insects’ adaptation to various host plants.

Other aphid species than *Megoura viciae* and *Acyrthosiphon pisum*, were not screened for Ca^2+^-binding proteins in their watery saliva. A Ca^2+^-binding protein was also identified in saliva of the green rice leafhopper; that this insect’s saliva was secreted into sieve tubes may indicate the presence of comparable mechanisms in different groups of phloem-feeding insects (Hattori et al., 2012). Previously, Hao et al. (2008) demonstrated that the brown planthopper *Nilaparvata lugens* activates callose synthases during plant infestation but is able to unplug sieve pores by activating β-1,3-glucanases. Whether aphids also influence filament formation of SEO proteins (Batailler et al., 2012) or influence callose degradation is currently unknown.

### INTERACTION WITH CHEMICAL DEFENSE

Several detoxifying proteins in the saliva of aphids were identified by enzymatic essays and novel approaches, including mass spectrometry. The detoxification of phenols by the secretion of polyphenoloxidase and peroxidase was reported for *Sitobion avenae* (Urbanska et al., 1998). The degradation of hydrogen peroxide could most likely interfere with defense signaling because hydrogen peroxide represents an activator of Ca^2+^ channels in the plasma membrane (Lecourieux et al., 2006).

Proteins that appear to interact directly with plant defense signaling are glucose dehydrogenase and glucose oxidase that were detected in the aphid species *Myzus persicae* and *Acyrthosiphon pisum* (Harmel et al., 2008; Carolan et al., 2011). Both potentially interfere with jasmonic acid (JA)-regulated defense responses that were shown to be induced during infestation of *Arabidopsis* by *Brevicoryne brassicae* (Kusnierczyk et al., 2011). Takemoto et al. (2013) noticed that endogenous JA production was less for *Acyrthosiphon pisum* infested broad bean plants. Furthermore, aphids appear to be able to modulate genes in the salicylic acid (SA) pathway (Zhu-Salzman et al., 2004). Cross-talk between JA and SA defense pathways (Pieterse et al., 2012) may allow aphids to suppress specific plant defense responses as has been previously described for whiteflies by Zarate et al. (2007). The role of SA and JA in plant-aphid interaction is reviewed by Louis and Shah (2013).

### SALIVARY EFFECTORS

Effectors are defined as proteins and/or small molecules that modify cell structure and function inside the host of a pathogen (Hogenhout et al., 2009). Aphid species were shown to pursue similar strategies and secrete effectors as components of their saliva (**Figure 1C**). Effectors in saliva were first shown for *Megoura viciae* by identifying proteins that bind Ca^2^^+^ (Will et al., 2007). Later it was shown that C002, a salivary protein that is secreted into the sieve tubes by *Acyrthosiphon pisum*, plays an important role in aphid feeding (Mutti et al., 2008). If C002 is silenced by RNA interference (RNAi) in *Acyrthosiphon pisum*, aphids’ life spans are reduced because they have problems reaching the sieve tubes and are thus unable to sustain ingestion (Mutti et al., 2006, 2008). Silencing of C002 homolog in *Myzus persicae* by feeding on transgenic plants showed lower aphid reproduction rates than usual but no overall change in survival rates (Pitino et al., 2011). When *Myzus persicae* fed on MpC002-expressing plants, an enhanced fecundity was observed (Bos et al., 2010), while the reproduction rates of *Myzus persicae* feeding on plants that express C002 from *Acyrthosiphon pisum* are not influenced (Pitino and Hogenhout, 2013). Further effectors with beneficial effect on aphid reproduction and thus on colonization are PIntO1 and PIntO2. Orthologs of C002, PIntO1and PIntO2 were detected in salivary gland transcriptome of multiple aphid species and appear to be specific for the respective aphid species (Pitino and Hogenhout, 2013). A recent study of the effector Me23 shows that effectors are specific not only to aphid but also to plant by demonstrating that the fecundity of *Macrosiphon euphorbiae* was enhanced when aphids fed on Me23 expressing *Nicotiana benthamiana* and not on Me23 expressing tomato (Atamian et al., 2013). Above described effectors may be able to facilitate ingestion by suppressing plant defense responses, perhaps by interfering with signal cascades as described for different fungi (reviewed in Stergiopoulos and de Wit, 2009) and appear to contribute to aphid-plant compatibility (Pitino and Hogenhout, 2013). In addition to effectors that promote aphid colonization, some effectors induce plant defense responses. MP10 and MP42 were shown to reduce fecundity when expressed in plants (Bos et al., 2010), possibly interacting with plant receptors of the NBS-LRR superfamily and thus triggering plant defense responses (Hogenhout and Bos, 2011). The identified aphid resistance genes *Mi-1.2 *in tomato (Martinez de Ilarduya et al., 2003) and *Vat* in melon (Dogimont et al., 2008) belong to the NBS-LRR receptor family (reviewed in Smith and Clement, 2012).

## PHLOEM-LOCATED APHID CONTROL STRATEGIES

As described, plant defense can be overwhelmed by aphids. For this reason, e.g., agro-biotechnological control strategies support plant defense by inserting additional insecticidal compounds into the sieve tubes. Approaches are the expression of PIs and antimicrobial peptides (AMPs) that naturally do not belong to the target plants defense system (reviewed in Will and Vilcinskas, 2013). PIs can be used to target proteases detected in the watery saliva (Carolan et al., 2009) and alimentary tract (Rahbé et al., 1995; Cristofoletti et al., 2003, 2006) of aphids; there the PIs may prevent the digestion of proteins within the sieve-tube sap (**Table 1**). Although for a long time researchers did not believe that aphids were able to use proteins as a source of nutrition, new findings show that aphids can digest proteins in sieve-tube sap (Pyati et al., 2011). The use of AMPs represents an approach that targets the endosymbiotic bacteria of aphids, assuming that disrupting these bacteria would negatively affect aphid fitness (Douglas, 2007). The primary (obligate) endosymbiotic bacteria *Buchnera aphidicola* (Baumann et al., 1995) improves the quality of aphid diet by supplying it with essential amino acids (IAGC, 2010) that are absent in sieve-tube sap (Gündüz and Douglas, 2009). Other endosymbiotic bacteria improve aphid fitness by giving resistance to pathogenic fungi (e.g., Lukasik et al., 2012) or increasing thermo tolerance (e.g., Russell and Moran, 2006). Although described methods of controlling aphids address different levels of interaction of pests and their respective host plants, a common goal is disrupting plant accession and nutrition uptake. Expressing defense agents in the sieve tubes is an effective way of accomplishing this disruption. Defense agents negatively affect fitness parameters (e.g., Le-Feuvre et al., 2007; Mutti et al., 2008; Pyati et al., 2011), such as growth, reproduction, and survival, which may reduce infestations among plants.

## Conflict of Interest Statement

The authors declare that the research was conducted in the absence of any commercial or financial relationships that could be construed as a potential conflict of interest.
